# An Extraordinary Case of Adhesion

**Published:** 1865-11

**Authors:** Wm. Meacher

**Affiliations:** Pardeeville, Wisconsin


### EDITORIAL AND MISCELLANEOUS.

### AN EXTRAORDINARY CASE OF ADHESION.

By WM. MEACHER, M. D., of Pardeeville, Wisconsin.

About a year ago Mr. Kinney, of Marcellon, came to me to have his thumb dressed, having got it injured while hitching a yoke of oxen to a wagon, by getting it caught in the chain.

Upon examining it, I found that a large piece of flesh had been jammed off, exposing the end and under surface of the bone. The piece of flesh fell on the ground, and Mr. Kinney picked it up, (thinking it a good plan to "save the pieces,") and replaced it, wrapping the thumb up with a bit of rag—in which condition it remained until I saw it, about two hours after the injury.

After looking at it, I hardly knew what to do with it, for it appeared evident that if I removed the piece, (which I did not do at all,) and cut off the end of the bone, I could not get flesh enough to cover the stump. Neither did I think I could get flesh enough to cover the stump, by amputating at the first joint; for the piece that came off reached half way to the second joint, and the patient objected to having it amputated above the joint. So, hardly knowing what else to do, I secured the piece in its place with some small strips of adhesive plaster, and left it for the present.

It remained so until the next day, when I examined it again and found the piece apparently alive and healthy. So I

concluded to let it alone as long as it kept so, which I did until it grew fast, and sound.

The Princeps Pollicis artery was torn off opposite the joint, and drawn out some; and when the piece of flesh was replaced the end was left sticking out at the side of the thumb, and I was obliged to touch it with caustic to stop its bleeding.

The patient was over 60 years old. The piece was not cut off, but jammed, or pinched off, and imperfectly replaced, or in other words, coaptation was imperfect.

---

We publish with pleasure the article of our esteemed correspondent, yet are compelled to say, that what it narrates is almost too much for our credulity, and makes us think that the patient has misled his surgeon—and possibly himself, into the supposition that the part was actually severed from all vascular connection with the body. This is so unlike what we every day observe, that we could only believe such a thing possible after undoubted experimental demonstration. As *apropos* to the subject, however, and tending to the credibility of the case reported by Dr. Meacher, we quote from Abernethy's Lectures on the Physiological views of John Hunter:

"Mr. Hunter was convinced that life might remain in a dormant state, in detached parts, for sixty hours. He therefore could not wonder at the facts with respect to transplantation or engrafting of portions of animal bodies with which he was acquainted; yet he says that the transplanted part must have life, to accept of the union, because he believed, that a correspondent and co-operating action was necessary for its accomplishment.

"Mr. Hunter observing how firmly the gum sometimes attached itself to a transplanted tooth, and thinking the comb of a cock resembled the gum in its texture, transplanted the tooth of a dog and set it in the comb, where it became firmly fixed. He next transplanted a gland taken from the abdomen of a cock, to a similar situation in the belly of a hen, where it also became attached, and as he believed, nourished, for he probably thought he had injected it from the general arterious system. The uniting medium by which it is firmly connected, is certainly very vascular, yet I do not see that any injection has passed into the vessels of the transplanted gland. The preparation is in the Museum, so that you can examine it for yourselves. Mr. Hunter probably believed that it was nourished, from its neither wasting nor decaying. As, however, the evidence was not very distinct, he next transposed the spur of a young hen, to the leg of a cock, and that of the latter, to the leg of the former bird, which spurs grew, and thus set the subject at rest in his mind. It may seem, however, curious, that the hen's spur grew to a greater size on the cock's leg, than it would have done upon the parent animal, which Mr. Hunter considered as a proof of the greater vigor of constitution of the male bird."

